# Primary Mucinous Adenocarcinoma of Appendix Invading Urinary Bladder with a Fistula: A Case Report

**DOI:** 10.1515/biol-2019-0064

**Published:** 2019-12-31

**Authors:** Jiwei Yang, Jianning Wang, Tongyi Men, Xiaoming Zhang, Xianduo Li, Bin Shen, Ping Zhou

**Affiliations:** 1 Department of Urology, The First Affiliated Hospital of Shandong First Medical University, No. 16766, Jingshi Road, Jinan, 250014, China; 2 Department of Pathology,The First Affiliated Hospital of Shandong First Medical University, Jinan 250014, China

**Keywords:** Adenocarcinoma, appendiceal carcinoma, fistula, bladder

## Abstract

An adenocarcinoma of the appendix invading the urinary bladder, which is difficult to be diagnosed before the operation, is an extremely rare disease. Only a few cases have been reported. Here we reported a case of patient diagnosed with the mucinous adenocarcinoma of the appendix invading the urinary bladder. The case reported in this study was a 54-years old man who was admitted due to a 6-month history of intermittent episodes of irritative voiding symptoms of the bladder, and weight loss. The patient did not have any gastrointestinal symptoms. The physical examination, laboratory examination, cytology of the urine, computed tomography and cystoscopy were inconclusive. The partial cystectomy, subsequent exploratory laparotomy and intraoperative frozen analysis revealed the appendiceal mucinous adenocarcinoma with a fistula to the urinary bladder. The appendectomy and the right hemicolectomy with a ileocolic anastomosis, the lymphadenectomy and the partial cystectomy limited to the anterior wall was performed. Six months after operation, the patient was in a good health with no obvious discomfort, no recurrence or distant metastases. The recommended treatment for the adenocarcinoma of the appendix invading the bladder with a fistula formation is as follows: appendectomy, right hemicolectomy with ileocolic anastomosis, lymphadenectomy, partial cystectomy and intraperitoneal hyperthermic chemoperfusion.

## 1 Introduction

The primary appendiceal carcinoma is a rare disease. It has an age-adjusted incidence of 0.12 cases per 1,000,000 people per year [1]. It accounts for 0.01%-1% of all gastrointestinal malignancies [2,3], and 4%-6% of appendiceal neoplasms [4]. It is found in only 0.08-0.2% of appendectomy specimens [5].

The international Classification of Diseases for Oncology group divides the adenocarcinoma of the appendix into three categories: colonic, mucinous and signet ring cell adenocarcinoma [1,6]. Among the three types, the signet ring type is extremely rare and the colonic type is the most common type. The mucinous type is usually well differentiated, often produces pseudomyxoma peritonei and is not metastatic until the late stage of the disease. The mean onset age of the mucinous type is between 50 and 60 years old, with a male to female ratio of 3-4:1 [7,8].

It is well known that tumors of the pelvic organs, such as the ovary, the rectum and the sigmoid colon, can invade the urinary bladder due to spatial contiguity. However, the appendiceal mucinous adenocarcinoma infiltrating the bladder is extremely rare and less than 20 cases have been reported [1]. The adenocarcinoma of the appendix invading the bladder with a fistula formation is even rarer. In our review of the literature, there were only 3 cases reported [8, 9, 10]. Here we reported a case of the adenocarcinoma of the appendix invading the urinary bladder and we reviewed the relevant literature.

## 2 Case report

**Figure 1:**
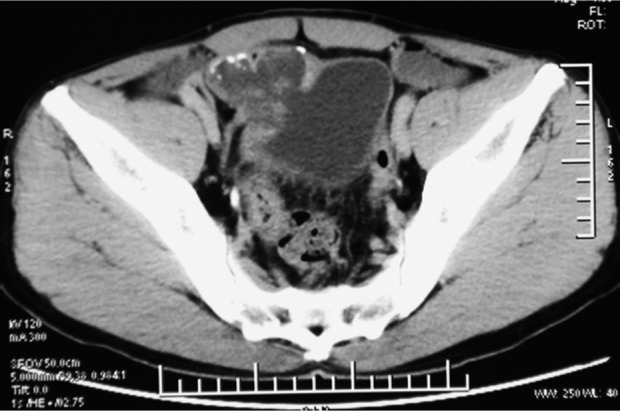
**CT scan shows an irregular slightly higher density**. The largest cross-section diameter was 5.2 cm×4.8 cm and its CT value was slightly higher than the intravesical CT value. There was some punctate calcification on the edge and inside the hyperdense area.

**Figure 2:**
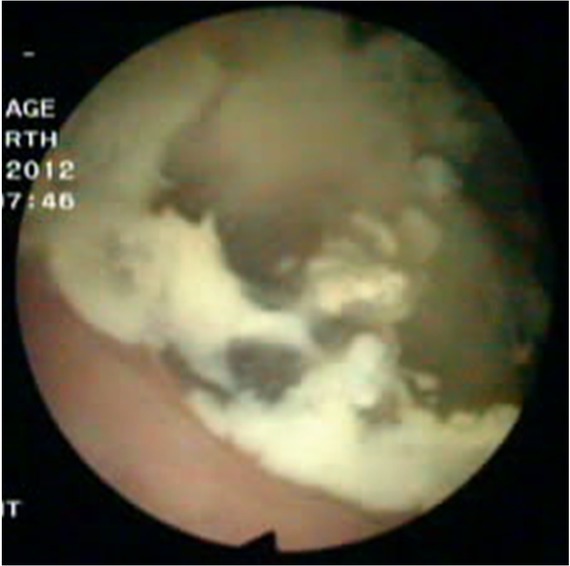
**Cystoscopy findings**. Cystoscopy was performed and an edematous broad opening with mucinous components in the right posterior wall of the bladder was observed.

A 54-years old man with a 6-month history of intermittent episodes of irritative voiding symptoms of the bladder and weight loss was admitted. The patient did not have any gastrointestinal symptoms such as pain, obstruction or melena, and he presented a negative medical history for gastrointestinal diseases. The physical examination was negative. An urine analysis revealed 30 white blood cells and 12 red blood cells per high power field. No malignant cells were observed in the urine. The carcinoembryonic antigen level was normal. The enhanced pelvic computed tomography (CT) scan revealed an irregular and slightly higher density (Figure 1), with the largest cross-section of 5.2 cm×4.8 cm. Its CT value was slightly higher than the intravesical CT value. There was some punctate calcification on the edge and inside the hyperdense area. A cystoscopy showed an edematous broad opening with mucinous components in the right posterior wall of the bladder (Figure 2).

The partial cystectomy was therefore performed, and the right posterior wall of the bladder was adherent to the peritonea. It was found, during the subsequent exploratory laparotomy, that the tumor was located between the ileocecum and the bladder. The appendix was not observed. The ileocecum firmly adhered to the bladder posterior wall with a fistula, which was full of mucus. The intraoperative frozen analysis revealed appendiceal mucinous adenocarcinoma. Thus, the mucinous adenocarcinoma of the appendix with a fistula to the urinary bladder was diagnosed. The following procedures: appendectomy, right hemicolectomy with ileocolic anastomosis, lymphadenectomy, and partial cystectomy limited to the anterior wall, were performed.

**Figure 3:**
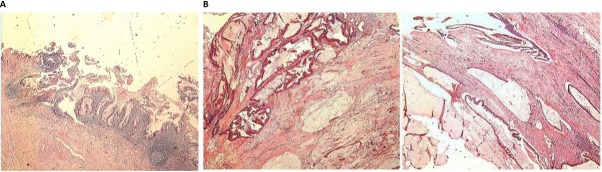
**Pathological morphology by HE staining**. Postoperative pathological examination confirmed the diagnosis of mucinous adenocarcinoma of the appendix (A). The entire bladder was filled with mucinous adenocarcinoma (B).

On a gross examination, accumulation of mucus within in the lumen and thickening of the wall were observed. A pathological examination confirmed a mucinous adenocarcinoma of the appendix with a transmural growth to the right posterior wall of the bladder (Figure 3). The lymph node metastasis was not observed. A pathological staging was pT4N0M0 (the TNM classification), and stage B (Ducks, Astler and Coller classification). The patient was discharged on the 15th postoperative day. He did not received chemotherapy due to economical reason. No obvious discomfort, no recurrence or distant metastases were observed in postoperative outpatient periodic review for 1 year.

**Informed consent**: Informed consent has been obtained from all individuals included in this study.

**Ethical approval**: The research related to human use has been complied with all the relevant national regulations, institutional policies and in accordance the tenets of the Helsinki Declaration, and has been approved by the authors’ institutional review board or equivalent committee

## 3 Discussion

The adenocarcinoma of the appendix invading the urinary bladder is an extremely rare disease. To our best knowledge, only a few cases have been reported in the world [1,3], and this disease is very difficult to be diagnosed before operation.

An appendicovesical fistula accounts for 5% of intestinal vesical fistula [11], and the most often cause is a delayed treatment of an acute appendicitis. The peak onset age of appendicovesical fistula is between the ages of 10 to 60, but the mean one age of appendiceal neoplasm invading the bladder, which occurs more often in males, is older [8].

An appendiceal neoplasm most often presents as an acute appendicitis or as a palpable abdominal mass. The mucinous adenocarcinoma invading the bladder does not have classical symptoms of gastrointestinal tracts and vesicoenteric fistula, such as abdominal pain and pneumaturia. Only irritative symptoms of the bladder or the gross hematuria are manifested. Therefore, it is difficult to make an early and accurate preoperative diagnosis of the appendicovesical fistula.

The key to diagnosis is the awareness and the vigilance for this disease. When the thickened appendix full of mucus was found during surgery, the mucinous adenocarcinoma invading the bladder should be considered, and the intraoperative frozen biopsy should be immediately performed. If the diagnosis is clear, the formal right hemicolectomy is considered as the traditional treatment for the patients with the non-metastatic adenocarcinoma of the appendix. The use of the appendectomy is limited to a small area of the appendix, especially for *in situ* and localized cases [12]. The 5-year survival rate was 20% with the appendectomy alone, while it was 45-63% with right hemicolectomy [13,14]. The mucinous adenocarcinoma has the tendency to produce conditions such as pseudomyxoma peritonei. In addition to the right hemicolectomy, the intraperitoneal chemotherapy or more accurate intraperitoneal hyperthermic chemoperfusion is recommended for mucinous adenocarcinoma of the appendix invading the bladder [12,15].

## 4 Conclusion

The adenocarcinoma of the appendix invading the urinary bladder is an extremely rare disease. Surgeons should be familiar with the necessary knowledge to make an appropriate and accurate diagnosis, which will lead to a prompt and correct treatment. The recommended treatment for the adenocarcinoma of the appendix invading the bladder with a fistula formation is as follows: appendectomy, right hemicolectomy with ileocolic anastomosis, lymphadenectomy, partial cystectomy and intraperitoneal hyperthermic chemoperfusion.
